# The synergism of *Clinacanthus nutans* Lindau extracts with gemcitabine: downregulation of anti-apoptotic markers in squamous pancreatic ductal adenocarcinoma

**DOI:** 10.1186/s12906-019-2663-9

**Published:** 2019-09-14

**Authors:** Ling-Wei Hii, Swee-Hua Erin Lim, Chee-Onn Leong, Swee-Yee Chin, Ngai-Paing Tan, Kok-Song Lai, Chun-Wai Mai

**Affiliations:** 1 Department of Life Sciences, School of Pharmacy, International Medical University, Kuala Lumpur, 57000 Malaysia; 2 Perdana University-Royal College of Surgeons in Ireland, Seri Kembangan, 43400 Selangor Malaysia; 3 Health Sciences Division, Abu Dhabi Women’s College, Higher Colleges of Technology, Abu Dhabi, United Arab Emirates; 4 Centre for Cancer and Stem Cells Research, Institute for Research Development and Innovation, International Medical University, Kuala Lumpur, 57000 Malaysia; 5 Department of Pharmaceutical Chemistry, School of Pharmacy, International Medical University, 57000 Kuala Lumpur, Malaysia; 6 Department of Land Management, Faculty of Agriculture, Universiti Putra Malaysia, Seri Kembangan, 43400 Selangor Malaysia; 7 Department of Cell and Molecular Biology, Faculty of Biotechnology and Biomolecular Sciences, Universiti Putra Malaysia, 43400 Seri Kembangan, Selangor Malaysia

**Keywords:** *Clinacanthus nutans*, Synergism, Pancreatic ductal adenocarcinoma, Gemcitabine

## Abstract

**Background:**

*Clinacanthus nutans* extracts have been consumed by the cancer patients with the hope that the extracts can kill cancers more effectively than conventional chemotherapies. Our previous study reported its anti-inflammatory effects were caused by inhibiting Toll-like Receptor-4 (TLR-4) activation. However, we are unsure of its anticancer effect, and its interaction with existing chemotherapy.

**Methods:**

We investigated the anti-proliferative efficacy of polar leaf extracts (LP), non-polar leaf extracts (LN), polar stem extract (SP) and non-polar stem extracts (SN) in human breast, colorectal, lung, endometrial, nasopharyngeal, and pancreatic cancer cells using 3-(4,5-dimethylthiazol-2-yl)-2,5-diphenyltetrazolium bromide, MTT assay. The most potent extracts was tested along with gemcitabine using our established drug combination analysis. The effect of the combinatory treatment in apoptosis were quantified using enzyme-linked immunosorbent assay (ELISA), Annexin V assay, antibody array and immunoblotting. Statistical significance was analysed using one-way analysis of variance (ANOVA) and post hoc Dunnett’s test. A *p*-value of less than 0.05 (*p* < 0.05) was considered statistical significance.

**Results:**

All extracts tested were not able to induce potent anti-proliferative effects. However, it was found that pancreatic ductal adenocarcinoma, PDAC (AsPC1, BxPC3 and SW1990) were the cell lines most sensitive cell lines to SN extracts. This is the first report of *C. nutans* SN extracts acting in synergy with gemcitabine, the first line chemotherapy for pancreatic cancer, as compared to conventional monotherapy. In the presence of SN extracts, we can reduce the dose of gemcitabine 2.38–5.28 folds but still maintain the effects of gemcitabine in PDAC. SN extracts potentiated the killing of gemcitabine in PDAC by apoptosis. Bax was upregulated while bcl-2, cIAP-2, and XIAP levels were downregulated in SW1990 and BxPC3 cells treated with gemcitabine and SN extracts. The synergism was independent of TLR-4 expression in pancreatic cancer cells.

**Conclusion:**

These results provide strong evidence of *C. nutans* extracts being inefficacious as monotherapy for cancer. Hence, it should not be used as a total substitution for any chemotherapy agents. However, SN extracts may synergise with gemcitabine in the anti-tumor mechanism.

**Electronic supplementary material:**

The online version of this article (10.1186/s12906-019-2663-9) contains supplementary material, which is available to authorized users.

## Background

Many cancer patients use therapies promoted as viable alternatives to conventional cancer treatment with questionable outcomes. Such unproven modalities can be potentially harmful. Furthermore, an even greater proportion of cancer patients use complementary therapies such as herbs and supplements along with conventional cancer treatment such as chemotherapy and radiation therapy. Some of these may have been proven to be adjunctive approaches that control symptoms and enhance quality of life. There is much controversy as to whether these natural health products should be taken during conventional cancer treatments and both sides of the divide provide valid arguments. More importantly, the drug-herb interaction effects of such complementary therapies with chemotherapy agents are often not studied during clinical trials or even receive post-marketing surveillance [1, 2]. Cancer development and progression is usually not driven by single cells. The tumor microenvironment drives the drug resistance and tumor survival [3–5]. It is hard to believe any single agent may effectively suppress cancer development and progression. Researchers have been actively targeting the Mother Nature to explore any potential regimen for cancer. Despite dietary or plant-isolated compounds [6–12] exhibiting a potent anticancer effect, thorough scientific investigation should be conducted in order to validate their effects on cancer treatment.

*Clinacanthus nutans*, or Sabah Snake Grass (SSG) as it is locally known in Malaysia, is a plant with indigenous origins in Southeast Asia, although its actual origin is unknown. It was originally isolated from Sabah, West Malaysia and hence, it is named after the location. The genus *Clinacanthus* consists of two species, *C. nutans* Lindau and *C. siamensis* Brem, with both belonging to the family Acanthaceae. *C. nutans*, a small shrub, is often cultivated and has long been used in Thailand as a traditional medicine for the treatment of skin lesions resulting in a *C. nutans* preparation for the relief of minor skin inflammation [13, 14]. Among cancer patients in Malaysia, SSG has been known to cure the latter stages of liver cancer; however, its consumption is advised to be carried out only following conventional treatments of chemotherapy and radiotherapy due to possible adverse effects that could arise. To the best of our knowledge, this claim has no scientific evidence to support it, and is made purely due to the cautioning of concomitant use of chemotherapy agents with other unproven agents. Several isolated studies have therefore investigated the claims. It was suggested the methanolic extracts of *C. nutans* had effects on human lung cancer (NCI-H23), cervical cancer (HeLa), liver cancer (HepG2), leukemia (K-562, Raji), neuroblastoma (IMR32), gastric cancer (SNU-1) and colon cancer (LS-174 T) cells. However the most active extract, chloroform extracts exhibited only a very low potency (IC_50_ = 47.31–47.70 μg/mL) against cancer cells [15]. The criteria established by the American National Cancer Institute for a crude extract to be considered as a potential cytotoxicity agent, it would achieve an IC_50_ less than 30 μg/mL when tested against a cell line. In another study, however, *C. nutans* methanolic extracts showed no significant cytotoxicity until at the highest concentrations tested under normoxic conditions [16]. Furthermore, *C. nutans* extracts tested against cyclophosphamide against COR-L23 cancer cell line with and without microsomal incubation did not show a significant (*p* > 0.05) reduction in IC_50_ values [17]. Thus, it is rather challenging for clinicians to recommend or to discourage the use of *C. nutans* in achieving the desired therapeutic outcomes. The need to standardise the experimental procedures, including using the standardised extracts, and to use a standardised in vitro anticancer procedure, is of the utmost importance to mitigating the anticancer potential of *C. nutans.*

In our previous study, we have prepared the standardised polar and non-polar fractions of *C. nutans* leaves and stem. These extracts were found to exhibit anti-inflammatory properties through inhibiting Toll-like Receptor 4 (TLR-4) activation and nitric oxide production, one of the key inflammatory mediators. The total phenolic contents and total flavonoid contents were correlated with its anti-inflammatory potency. The polar leaf extracts were also found to inhibit the hallmark inflammatory mediators, such as p65, p38, pERK, pJNK and pIRF3. More importantly, we have established that these standardised bioactive extracts of *C. nutans* had no cytotoxicity on human embryonic kidney cells and macrophages [18]. In this study, we aimed to expand our knowledge by investigating the anticancer effects of these standardised *C. nutans* leaves and stem in human cancer cells. Since most patients are likely also to take both chemotherapy agents and *C. nutans* concomitantly*,* we also investigated the interaction between chemotherapy agents and *C. nutans.* The current investigation was also designed to determine the possible cell death behind the interaction between *C. nutans* extracts and gemcitabine in pancreatic cancer cells.

## Methods

### Preparation of plant extracts

As established in the previous study [18], the *C. nutans* plant was identified by a botanist from the Forest Research Institute of Malaysia, in an orchard at Pahang, Malaysia. The voucher specimens of the plant were deposited in the Malaysian Agricultural Research and Development Institute herbarium with the specimen numbers MDI 12807 and MDI 12808. *Clinacanthus nutans* polar leaf extracts (LP), non-polar leaf extracts (LN), polar stem extract (SP) and non-polar stem extracts (SN) were prepared based on the previous study without modification [18]. The leaves and stem bark were dried and turned to powder separately before extraction. The dried powders were immersed in polar solvents (methanol and dichloromethane) or non-polar solvents (hexane and diethyl ether) for 3 days at room temperature. The preparation was in accordance to the way the extracts were normally consumed by the public and from methods described in published literature. LP, SP, LN and SN were prepared, and the solvents were removed under vacuum using a rotary evaporator at 60 °C. We routinely determined the total phenolic content (TPC) and total flavonoid content (TFC) of extracts using standardized assays as per described in previous studies. Only dried extracts in which the TPC and TFC were within a 5% variation from the previous published findings [18], were then subjected to further experiments. All chemical reagents were purchased from Sigma-Aldrich (St Louis, MO, USA) unless specified.

### Cell lines and cell culture

Cancer cells derived from cancers that had high prevalence or high mortality were selected for this study. All cells were obtained from the American Tissue Culture Collection (Rockville, MD, USA), unless specified. The selected cells were human breast cancer cells (MCF7, MDA-MB-231, MDA-MB-468, HCC38), colon cancer cells (HCT116, HT29, SW48, Caco2), lung cancer cells (A549, NCI-H1299, NCI-H23, Calu-1), endometrial cancer cells (AN3-CA, HEC-1-A, HEC-1-B, RL95–2), nasopharyngeal cancer cells (CNE1, HK1, SUNE1, TWO1), and human pancreatic ductal adenoma, PDAC (AsPC1, BxPC3, SW1990, Panc 10.05). Human nasopharyngeal cancer cells were shared with us by Professor Sam C. K, University of Malaya, Malaysia. Unless specified, all cells were cultured in RPMI-1640 supplemented with 10% fetal bovine serum, 100 IU/mL of penicillin, 100 μg/mL of streptomycin, with L-glutamine, 4.5 g/L glucose and sodium pyruvate as per established in previous studies [9, 11, 19–23]. Instead of RPMI-1640, Calu-1 were cultured in Dulbecco’s Modified Eagle’s Medium (DMEM); RL95–2 were cultured in DMEM:F12; AN3CA and HEC-1B were cultured in Eagle’s Minimum Essential Medium; HEC-1A were cultured in McCoy’s 5A media. All cells were maintained at 37 °C under 5% carbon dioxide in a humidified incubator.

### Cytotoxicity assay

Since the previous study [18] established that the extracts at 100 μg/mL did not induce significant cytotoxicity on human embryonic kidney cells and macrophage, this indicated that the extracts may not have any cytotoxic effect to non-cancerous cells. Hence, concentrations used were up to 100 μg/mL. Cells were plated in 384-well plates for 24 h followed by LP, LN, SP and SN treatment at 1-100 μg/mL for 72 h incubation as per established in previous studies [11]. As a comparison, we also tested vitexin and isovitexin (Sigma Aldrich, USA), the pure compounds that were commonly isolated *Clinacanthus nutans* extracts [13]. CellTitre-Glo® Luminescent Cell Viability Assay kit (Promega, USA) was used to quantify the cell viability based on the luminescence readings recorded using SpectraMax M3 Multi-Mode Microplate Reader (Radnor, USA). IC_50_ values were calculated based on the dose-response curve generated. The experiments were validated using with 100 μg/mL gemcitabine and 5-fluorouracil, the first line clinically used anti-cancer agents.

### Gemcitabine combination analysis

The drug combination analysis was performed using the method developed by Chou and Talalay [24]. Multiple fixed-ratio dose–effect curves and the calculations of combination index (CI) and drug reduction index (DRI) were generated using Calcusyn 2.1 software (Biosoft, Cambridge, UK) as previously described [24–27]. CI values of < 1, = 1, and > 1 respectively indicate synergism, additive effect, and antagonism (Additional file 1: Table S1). DRI values were used to describe the dose reduction potential of the combination by predicting the dose of gemcitabine and SSG needed when used in combination to achieve a defined effect level (fraction affected, Fa) in comparison with the single-agent dose required for such effect. In principle, the dose reduction potential with DRI > 1 can be clinically valuable in reducing the risk of developing drug toxicity toward the host while retaining the therapeutic efficacy in a synergistic drug combination [28].

Since we could not exclude the possibility that SN extracts and gemcitabine might act simultaneously through different mechanisms, we also included the Bliss independence model to define the combinatorial effects. To further determine the combination effects using Highest Single Agent (HSA) model and Bliss independence model as previously described, Combenefit® software (Cancer Research UK Cambridge Institute, United Kingdom) was employed for drug interaction analysis [25, 29–31]. The volume of Bliss interaction for dose-response combination matrix was statistically evaluated and graded accordingly by MacSynergy II program version 1.0, in which volume values of below -25 μM^2^%, between − 25 and 25μM^2^%, and above 25μM^2^% were respectively considered Bliss antagonism, Bliss additivity and Bliss synergism (Additional file 1: Table S2) [32].

### Quantification of TLR-4 levels

Our previous study [18] suggested that SN extracts inhibited LPS induced TLR4 activation in RAW264.7 cells, murine macrophage. In order to investigate whether the TLR4 inhibitory effect of SN extracts is related to the synergistic anti-cancer effects of SN extracts and gemcitabine on pancreatic cancer cells, cells were treated for 72 h and the protein lysates were quantified by sandwich enzyme immunoassay principle using the TLR-4 enzyme-linked immunosorbent assay (ELISA) kit (Biocompare, Germany) as described in the manufacturer’s instructions. Cancer cells treated with 0.1% dimethylsulfoxide (DMSO) were used as negative control in this experiment.

### Determination of the mode of cancer cell death

In order to quantify the degree of cell death induced by the gemcitabine and/or SN extracts, pancreatic cancer cells were treated for 72 h and the nucleosomes were quantified by sandwich enzyme immunoassay principle using the Cell Death Detection ELISA^PLUS^ kit (Roche Diagnostics, Indianapolis, U.S.A.) as described in the manufacturer’s instruction and in previous study [20]. The enrichment factors of apoptosis were calculated based on the absorbance of cells treated with compound or combination over absorbance of cells treated with vehicle control (0.1% DMSO).

### Detection of apoptosis by Annexin V flow cytometry

PE Annexin V Apoptosis Detection Kit (BD Biosciences, USA) was used to quantify the apoptotic cell population, as described previously (10–11). Both floating and attached pancreatic cells were collected 72 h after the treatment with gemcitabine and/or SN extracts. All cells were analyzed using a BD FACS Calibur flow cytometer and the BD CellQuest Pro Software (version 5.1.1; BD Biosciences, USA) as described previously (11).

### Apoptotic antibody array

With the aim of identifying the cellular apoptotic markers involved in pancreatic cancer cells treated with gemcitabine and SN extracts, cells were treated with gemcitabine and/or SN for 72 h followed by plating the cell lysates into RayBio® Human Apoptosis Antibody Array (RayBiotech, USA) All reagents and chemicals were supplied by the manufacturer and conducted as per our previous study [22]. The fold changes were calculated after being normalised with cells treated with 0.1% DMSO.

### Protein isolation and immunoblotting

Protein lysates of pancreatic cancer cells treated with gemcitabine and/or SN for 48 h, were extracted in ice-cold lysis buffer (1% NP-40, 1 mM DTT and protease inhibitors in PBS), as described previously (10–11). Fifty micrograms of total protein was subjected to sodium dodecyl sulfate polyacrylamide gel electrophoresis followed by immunoblotting. Primary monoclonal antibodies against bax (D2E11), bcl-2 (D55G8), cIAP2 (58C7), XIAP (3B6) and anti-rabbit monoclonal antibodies were obtained from Cell Signaling, USA. Mouse monoclonal antibodies against and GAPDH (G-9; 1:1000) were obtained from Santa Cruz Biotechnology.

### Statistical analysis

All data was reported as mean ± standard deviation from a minimum of three independent experiments. Statistical significance was analysed using one-way analysis of variance (ANOVA) and post testing using Dunnett’s test through SPSS for Windows. A *p*-value of less than 0.05 (*p* < 0.05) was considered significantly different compared to negative control, treatment with 0.1% DMSO.

## Results

### *C. nutans* extracts selectively inhibit proliferation of human pancreatic ductal adenocarcinoma

To evaluate whether *C. nutans* extracts exhibited anti-proliferative effects, a total of 23 human cancer cells (MCF7, MDA-MB-231, MDA-MB-468, HCC38, HCT116, HT29, SW48, Caco2, A549, NCI-H1299, NCI-H23, Calu-1, AN3CA, HEC-1-A, HEC-1-B, RL95–2, CNE1, HK1, SUNE1, TWO1, AsPC1, BxPC3 and SW1990) were treated with 1-100 μg/mL of LP, LN, SP or SN for 72 h. SN possess the most potent (IC_50_: 31.21–39.12 μg/mL) and selective anti-proliferative effects towards human pancreatic cancer cells (AsPC1, BxPC3, and SW1990), sparing the human non-cancer cells (MCF10A, ARPE19, MRC5 and CCD841 CoN). As shown in Table 1, the lowest IC_50_ results were found among human pancreatic cancer cells treated with SN extracts (IC_50_: 31.21–39.12 μg/mL). Vitexin and isovitexin, the most commonly isolated *C. nutans* were not active (IC_50_ > 100 μg/mL) against human cancer cells.

**Table 1:** The IC_50_ of *C. nutans* Extracts on Cancer Cells

Cell Type	Cell Line	Vitexin	Isovitexin	LP	LN	SP	SN
Breast Cancer	MCF7	> 100	> 100	> 100	> 100	> 100	> 100
	MDA-MB-231	> 100	> 100	> 100	> 100	> 100	> 100
	MDA-MB-468	> 100	> 100	> 100	> 100	> 100	> 100
	HCC38	> 100	> 100	> 100	> 100	> 100	98.43 ± 1.51
Colorectal Cancer	HCT116	> 100	> 100	> 100	> 100	> 100	> 100
	HT29	> 100	> 100	> 100	> 100	> 100	> 100
	SW48	> 100	> 100	> 100	> 100	> 100	> 100
	Caco2	> 100	> 100	> 100	> 100	> 100	> 100
Lung Cancer	A549	> 100	> 100	> 100	> 100	> 100	71.11 ± 3.51
	NCI-H1299	> 100	> 100	> 100	> 100	> 100	> 100
	NCI-H23	> 100	> 100	> 100	> 100	82.28 ± 5.90	53.21 ± 1.33
	Calu-1	> 100	> 100	> 100	> 100	> 100	> 100
Endometrial Cancer	AN3CA	> 100	> 100	> 100	> 100	97.77 ± 1.12	50.51 ± 3.16
	HEC-1-A	> 100	> 100	> 100	> 100	> 100	> 100
	Hec-1-B	> 100	> 100	> 100	> 100	> 100	> 100
	RL95–2	> 100	> 100	> 100	> 100	> 100	> 100
Nasopharyngeal Cancer	CNE1	> 100	> 100	> 100	> 100	> 100	> 100
	HK1	> 100	> 100	> 100	> 100	> 100	> 100
	SUNE1	> 100	> 100	> 100	> 100	> 100	> 100
	TWO1	> 100	> 100	> 100	> 100	> 100	> 100
Pancreatic Cancer	BxPC3	> 100	> 100	> 100	> 100	80.11 ± 7.15	39.12 ± 3.35
	SW1990	> 100	> 100	> 100	> 100	82.21 ± 4.28	30.91 ± 3.16
	AsPC1	> 100	> 100	> 100	> 100	70.77 ± 3.21	31.21 ± 7.46
Non-cancerous Cells	MCF10A	> 100	> 100	> 100	> 100	> 100	> 100
	ARPE19	> 100	> 100	> 100	> 100	> 100	> 100
	MRC5	> 100	> 100	> 100	> 100	> 100	> 100
	CCD841 CoN	> 100	> 100	> 100	> 100	> 100	> 100

### Combination effects of SN extracts and gemcitabine on human pancreatic ductal adenocarcinoma

**Fig. 1:**
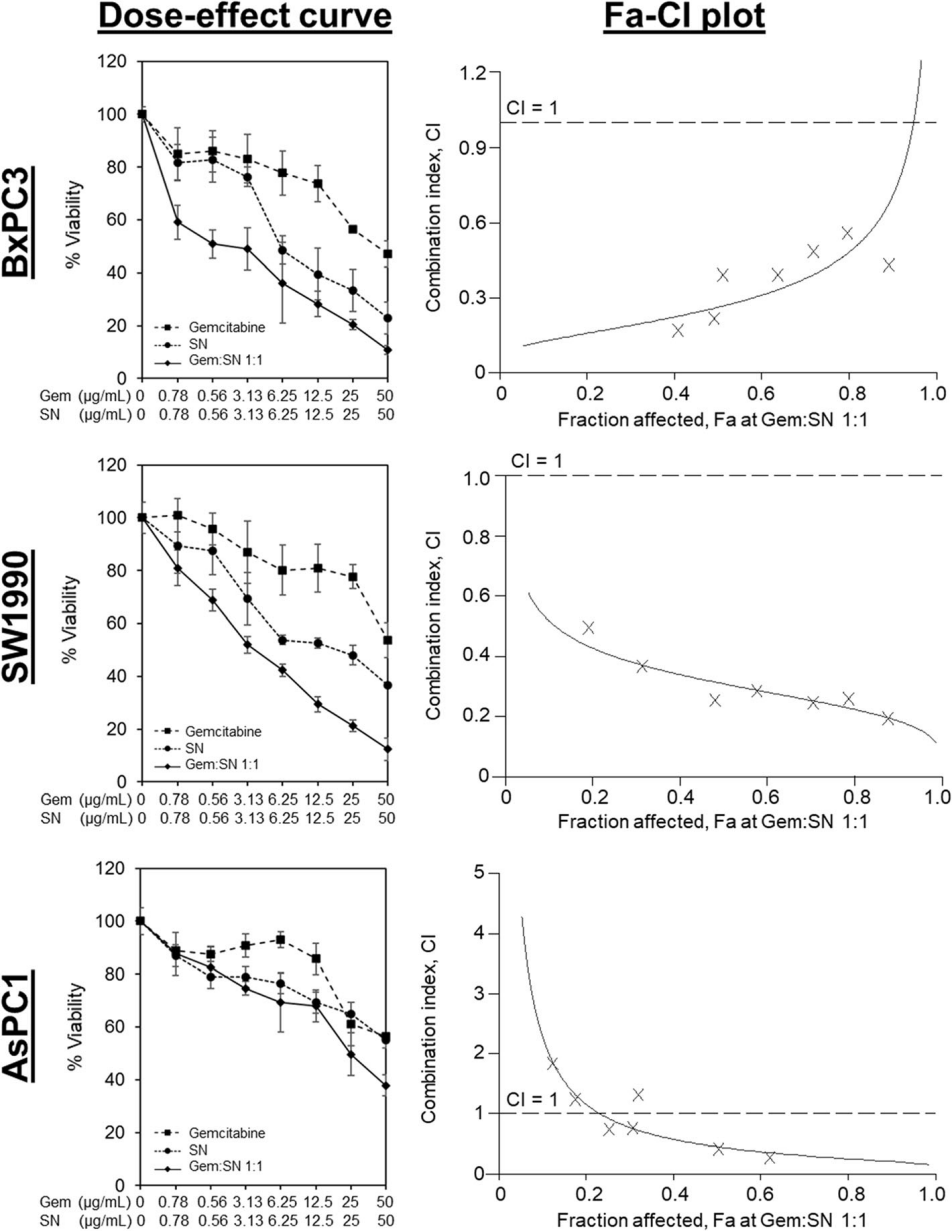
Synergistic effects of SN extracts and gemcitabine on PDAC. The 72 h treatment effects of SN and gemcitabine combination and single agent alone on PDAC cell viability were illustrated in the dose-effect curves. The treatment concentration ratio of SN to gemcitabine shown was 1:1. Each bar represents the mean ± standard deviation of three independent experiments. The curves in the Fa-CI plot showed the CI versus the fraction of PDAC cells that were inhibited by the combined treatment of SN and gemcitabine at concentration ratio of 1:1. The combinations were synergistic when CI values were < 1

**Fig. 2:**
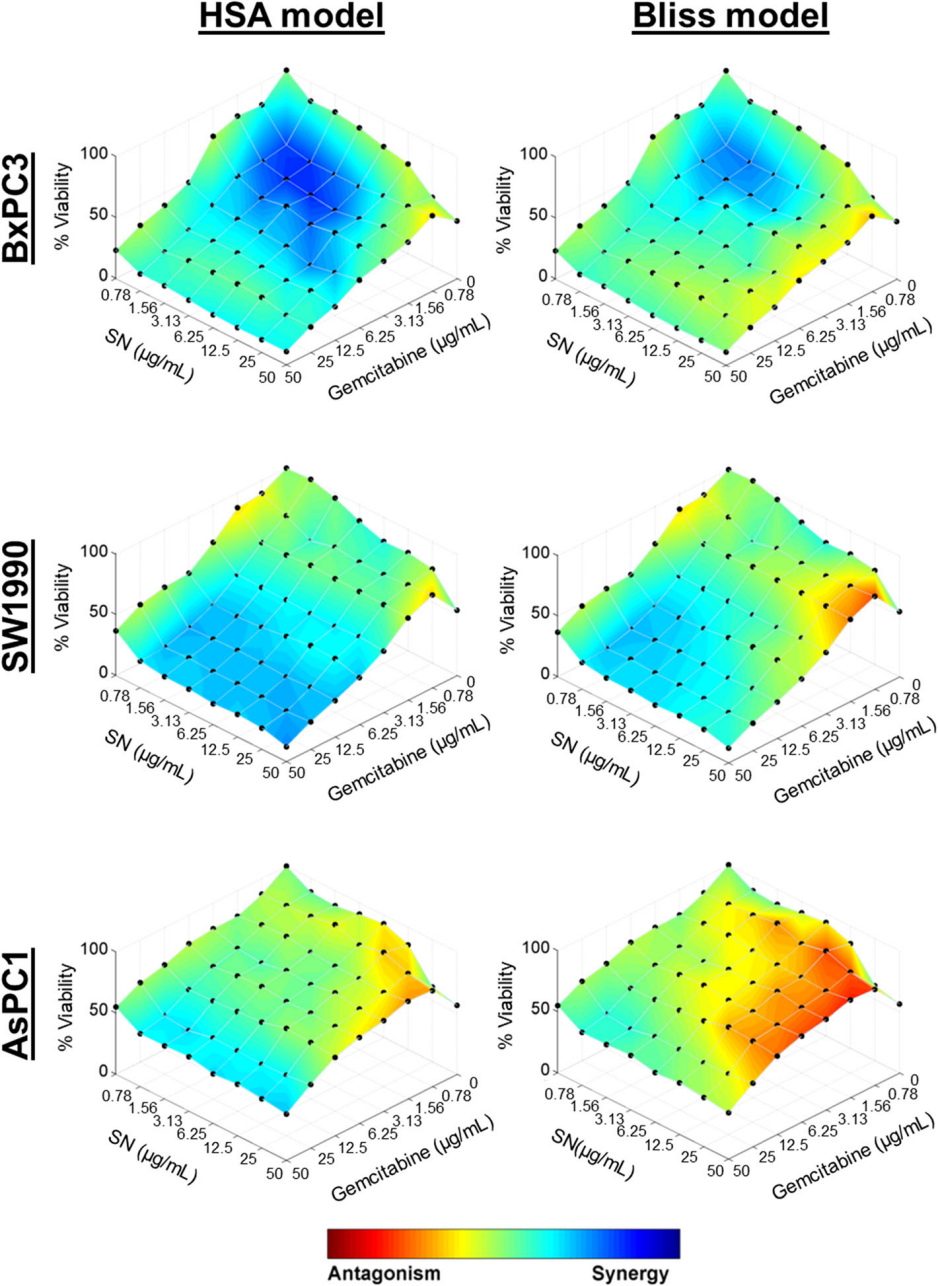
Combinatory effects of SN extracts and gemcitabine on PDAC. The efficacy of drug combinations was assessed by treating cells in an 8 × 8 matrix combination of SN and gemcitabine that was 2-fold serially diluted from the highest concentration of 50 mg/L. Cell proliferation was evaluated in 96-well plates using MTT assay at 72 h after treatment. Combenefit software was used to generate and assess the dose-response surface curves and drug interaction effects following the HSA model and Bliss independence model as implemented in the software. The level of synergy (blue) or antagonism (red) at each combination was indicated by the color scale shown. The net Bliss interaction volumes of the matrix drug combination were calculated based on MacSynergy II program and graded accordingly. All the experiments were conducted in triplicate and performed independently

To investigate whether there was synergism between SN extracts and gemcitabine, via enhancement of gemcitabine sensitivity in the pancreatic cancer cells, we evaluated the anti-proliferative effects of SN extracts in combination with gemcitabine in the squamous-like SW1990 and BxPC3 pancreatic cancer cells, and the progenitor-like AsPC1 pancreatic cancer cells. As shown in Fig. 1, SN extracts potentiated the anti-cancer effect of gemcitabine in both SW1990 and BxPC3, but not in AsPC1. The CI values and the combination analyses based on the HSA and Bliss independence models indicated that the combination of SN extracts and gemcitabine exerted significant synergism to inhibit the squamous subtype pancreatic cancer cells (SW1990 and BxPC3) being tested (Figs. 1 and 2, Table 2 and 3, Additional file 1: Figure S1, Additional file 1: Table S2, and Additional file 1: Table S3). The combinatorial treatment of SN extracts and gemcitabine did exert Excess Over Highest Single Agent (EOHSA) of up to 19% at certain dose combinations tested in AsPC1, suggesting that SN extracts might enhance gemcitabine activity albeit in a narrow dose range which encourages further investigations (Additional file 1: Figure S1).

**Table 2:** Combination Index of Gemcitabine and SN Extracts Combinatorial Treatment at Different Ratios on PDAC

Cell Lines	Gem^a^:SN ratio	Combination Index (CI) Values at					Interaction
		ED50	ED75	ED90	Mean	±SD	
BxPC3	1:4	0.48	0.60	0.83	0.64	± 0.18	Synergism
	1:2	0.29	0.41	0.59	0.43	± 0.15	Synergism
	1:1	0.26	0.42	0.70	0.46	± 0.22	Synergism
	2:1	0.31	0.45	0.68	0.48	± 0.19	Synergism
	4:1	0.44	0.49	0.55	0.49	± 0.05	Synergism
SW1990	1:4	0.50	0.39	0.30	0.39	± 0.10	Synergism
	1:2	0.33	0.26	0.21	0.27	± 0.06	Strong Synergism
	1:1	0.31	0.24	0.19	0.24	± 0.06	Strong Synergism
	2:1	0.27	0.27	0.27	0.27	± 0.00	Strong Synergism
	4:1	0.20	0.28	0.38	0.29	± 0.09	Strong Synergism
AsPC1	1:4	1.12	0.87	0.74	0.91	± 0.19	Nearly Additive
	1:2	0.59	0.42	0.34	0.45	± 0.13	Synergism
	1:1	0.45	0.28	0.21	0.32	± 0.13	Synergism
	2:1	0.34	0.20	0.15	0.23	± 0.10	Strong Synergism
	4:1	0.30	0.17	0.12	0.20	± 0.09	Strong Synergism

^a^*Gem* Gemcitabine, *SN* SN Extracts

**Table 3:** Bliss Interaction Volume of Gemcitabine and SN Extracts Matrix Combination on PDAC

Cell Lines	Bliss Interaction Volume (μM^2^%)	Description
BxPC3	168.93	Strong Bliss synergy
SW1990	365.60	Strong Bliss synergy
AsPC1	− 368.61	Strong Bliss antagonism

In addition, based on the DRI assessments (Additional file 1: Table S3), it was estimated that the concentration of gemcitabine required to achieve its ED50, which is the median effective dose (fraction affected, Fa = 0.5), could be reduced by 2.38- to 5.28-fold when combined with SN extracts in all the cell lines being tested. Together, our results implied that synergistic interaction exists between SN extracts and gemcitabine, with SN extracts strongly enhancing gemcitabine sensitivity in squamous subtype of pancreatic cancer cells, and the favourable DRI trend obtained might be exploited as an added benefit from combining SN with gemcitabine in pancreatic cancer cells. Based on the findings above, a subsequent apoptotic study was conducted using freshly prepared 5 μg/mL of SN extracts and 5 μg/mL of gemcitabine (at 1:1 ratio) on human pancreatic cancer cells.

### SN extracts and gemcitabine synergistically induced apoptosis by downregulating anti-apoptotic bodies, independent of TLR-4 expression

**Fig. 3:**
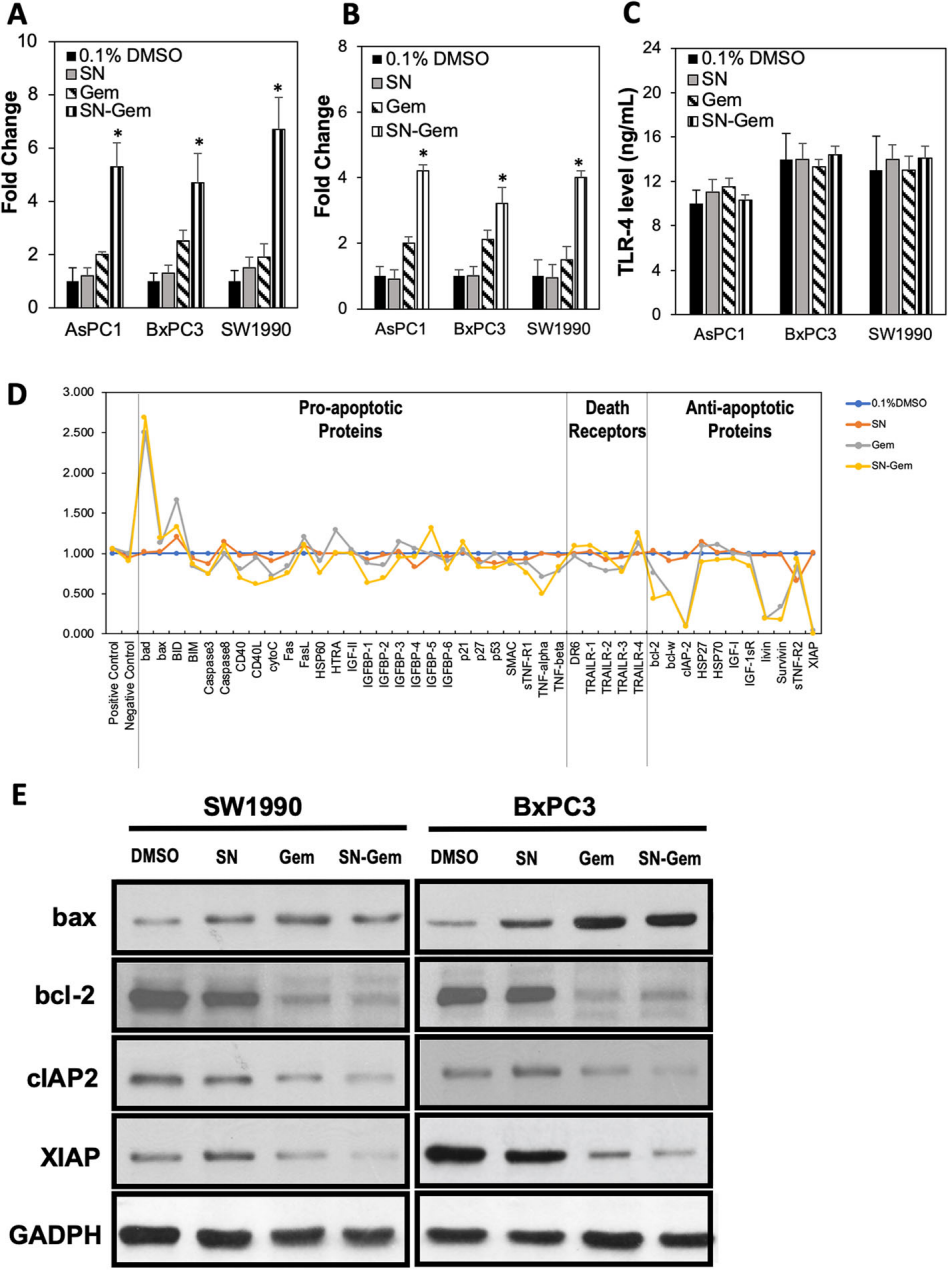
Apoptotic effects of SN extracts and gemcitabine on PDAC cells. **a** AsPC1, BxPC3 and SW1990 were treated with 0.1% DMSO, SN extracts, gemcitabine, or combination of both SN extracts and gemcitabine. The cells were then harvested for apoptotic induction using ELISA assay. **b** Apoptotic cell death in AsPC1, BxPC3 and SW1990 cells was quantified using Annexin V/7-AAD flow cytometry at 72 h following treatment. **c** The same lysates were tested for TLR-4 expression using ELISA assay. **d** SW1990 cells were treated with 0.1% DMSO, SN extracts, gemcitabine, or a combination of both SN extracts and gemcitabine, and the proteins were harvested for protein array analysis. **e** BxPC3 and SW1990 cells were exposed to 0.1% DMSO, SN extracts, gemcitabine, or a combination of both SN extracts and gemcitabine for 48 h. Protein lysates were subjected to SDS-PAGE. GAPDH was used as loading control. Each bar represents the mean ± standard deviation of three independent experiments

To determine the mode of cell deaths induced by the synergistic effects of SN extracts and gemcitabine, we quantified the apoptotic nucleosomes induced by 0.1% DMSO, 5 μg/mL SN extracts and/or 5 μg/mL gemcitabine. As shown in Fig. 3a, 5 μg/mL of SN and 5 μg/mL of gemcitabine (1:1 ratio) significantly induced more apoptotic nucleosomes in all pancreatic cancer cells (4.7- to 6.7-folds). SN extracts (5 μg/mL) only induced 1.2- to 1.5-folds of apoptotic nucleosomes while 5 μg/mL of gemcitabine only induced 1.9- to 2.5-folds of apoptotic nucleosomes. Combination of SN and gemcitabine also induced 3.4–4.2 fold of apoptosis in Annexin V assay. It was found to be much higher fold of apoptotic cells death as compared to apoptotic induction when pancreatic cancer cells were treated with 0.1% DMSO, SN, or gemcitabine.

Since a previous study [18] suggested that SN extracts inhibited TLR-4 expression and the TLR-4 activation in macrophages, we also investigated the effects of SN extracts and/or gemcitabine on TLR-4 expression in human pancreatic cancer cells. As shown in Fig. 3c, the TLR-4 expressions on AsPC1, BxPC3 and SW1990 were not significantly reduced by SN extracts, gemcitabine or the combination of both SN extracts and gemcitabine compared to cells treated with 0.1% DMSO. These results indicated that the synergistic effects of SN extracts and gemcitabine was independent to TLR-4 expression on human pancreatic cancer cells.

To further investigate the apoptotic cell death profiles induced by SN extracts and gemcitabine on human pancreatic cancer cells, SW1990 cells (the squamous pancreatic cancer cells with the highest apoptosis induction by SN and gemcitabine) were treated with 5 μg/mL SN extracts and/or 5 μg/mL gemcitabine and subjected to Raybio® Human Apoptosis Antibody Array (Norcoss, GA, USA). Our results showed that SW1990 cells treated with combination of SN extracts and gemcitabine (1:1), showed 2.69-fold and 1.333-fold increases of bax (bcl-2-associated X protein) and BID (Bcl-2 homology 3 interacting domain death agonist) respectively as compared to cells treated with 0.1%DMSO (Fig. 3d). Similarly, SW1990 cells treated with 5 μg/mL gemcitabine (but not 5 μg/mL SN extracts) showed 2.500-folds and 1.667-folds increases of bax and BID respectively. Gemcitabine reduced the expression of anti-apoptotic bodies, namely bcl-2 (B-cell lymphoma 2), cIAP-2 (cellular inhibitor of apoptosis 2), livin, survivin and XIAP (X-linked inhibitor of apoptosis protein) in SW1990 cells. However, our results showed that the combination of SN extracts and gemcitabine had a greater reduction of these anti-apoptotic bodies expression in SW1990 cells, compared to cells treated with gemcitabine alone. The combination of SN and gemcitabine did not affect the expression of TNF (tumor necrosis factor) related apoptosis inducing ligand (TRAILR1–4), selected pro-apoptotic bodies and anti-apoptotic bodies. To confirm the combinatory effects on apoptosis pathways, we evaluated the protein levels of bax (pro-apoptotic), bcl-2, cIAP2 and XIAP (anti-apoptotic) in SW1990 and BxPC3 cells following treatement with SN extracts and gemcitabine. As highlighted in Fig. 3e, the combination of SN extracts and gemcitabine upregulated bax; but downregulated the anti-apoptotic proteins such as bcl-2, cIAP-2 and XIAP. The results were similar to findings observed in antibody array. These results suggest that synergistic apoptotic induction by upregulating bax and downregulating bcl-2, cIAP-2, and XIAP in human pancreatic cancer cells.

## Discussion

In recent years, the use of complementary and alternative medicine among cancer patients has been raising. This exponential increase could be attributed to the enhanced awareness of the general public to social media who are marketing the benefits of complementary medicine and the overall misgivings towards conventional cancer treatments. Furthermore, patients consume complementary and alternative medicine because they believe it will improve their quality of life. Some patients may even think complementary and alternative medicine can prolong life and promote cancer remission. These common complementary and alternative medicines include herbs, vitamins, minerals, homeopathy, naturopathy and specialized diets [33–36]. Numerous studies reported that 48–88% cancer patients used complementary and alternative medicine as part of their cancer therapy [37–39]. Despite the popularity of complementary and alternative medicine, there is limited research evaluating the scientific efficacy of complementary and alternative medicine in cancer treatment as well as its interaction with conventional cancer treatment. In addition, compared to patients receiving conventional cancer treatment, patients who chose complementary and alternative medicine also showed higher refusal rates of surgery, chemotherapy, radiotherapy, and hormone therapy. These factors lower the 5-year overall survival rates, inadvertently leading to greater mortality risks [36]. Hence, the need for conclusive evidence of the evidence of complementary and alternative medicine in cancer treatment is needed.

Based on folk medicine, *C. nutans*, or Sabah Snake Grass is one of the popular herbs and perceived to have anti-cancer effects [13–15]. To the best of our knowledge, only several isolated studies have thus far investigated the claims. Our previous study [18] suggested *C. nutans* extracts exhibit potential immunomodulator effects. Evidence suggests pancreatic cancer may susceptible to immunotherapies or immunodulators [40]. It is the interest of Asians to thoroughly investigate the role of *C. nutans* in cancer. The extracts were found to induce low anti-proliferative effect (IC_50_ = 47.31–47.70 μg/mL) against in vitro lung, cervical, liver, leukemia, gastric and cancer cells [15]. Our study has also shown that most of LP, LN, SP and SN extracts have very weak (IC_50_ > 30 μg/mL) anti-cancer effects when tested against 4 breast cancer cells, 4 colorectal cancer cells, 4 lung cancer cells, 4 endometrial cancer cells, 4 nasopharyngeal cancer cells and 3 pancreatic cancer cells, suggesting *C. nutans* may have no or very week anti-proliferative effect in cancer cells. The most potent *C. nutans* extracts amongst all tested extracts was SN extracts, with IC_50_ around 30.91–39.12 μg/mL on SW1990, AsPC1 and BxPC3. There were extensive phytochemistry studies, isolating many compounds from *C. nutans*, trying to investigate the anti-cancer effects of these isolated compounds. These flavonoids and terpenoids were also commonly isolated from various other plants [41, 42].

Previous study [42] published extensive metabolite profiling of *C. nutans* extracts. The research team identified betulin, lupeol and beta-sitosterol (Sigma Aldrich, USA) in stem extracts, which were not identified from other *C.nutans* extracts. Isovitexin, vitexin, rutin, chlorogenic acid and gallic acid (Sigma Aldrich, USA) were found in other extracts but not in SN extracts. In the attempt to correlate the bioactivity of these compounds to the observed anticancer effects of SN extracts, we purchased the pure compounds and tested it individually in our in vitro cytotoxicity assay. However, none of these compounds exhibited a promising anti-cancer effect against human pancreatic cancer cells (Additional file 1: Table S4). The observed anticancer effects may be due to novel compounds or a combinatory of compounds. To date, none of the isolated compounds or extracts have reached the patient as a promising anti-cancer agent. It is very unlikely a single isolated compound or a single extract from *C. nutans* alone may render a promising anticancer effects. Thus, it is not recommended to consume these extracts as a total replacement to conventional cancer chemotherapy.

According to the locals, many of the cancer patients were also taking the *C. nutans* extracts while receiving the chemotherapy and some of them claimed they have a ‘better recovery’. We were interested to find out whether the anti-cancer effects exhibited by chemotherapy may be enhanced or abolished in the present of extracts *C. nutans*. Thus, we then proceeded to investigate the effects of SN extracts (the most potent extracts) together with gemcitabine (the first line chemotherapy for pancreatic cancer patients) in both squamous pancreatic cancer cells (SW1990 and BxPC3) as well as progenitor pancreatic cancer cells (AsPC1). To our surprise, SN extracts strongly enhanced gemcitabine sensitivity in the squamous subtype of pancreatic cancer cells (SW1990 and BxPC3) but not the progenitor pancreatic cancer cells (AsPC1), and the favourable DRI trend obtained might also be exploited as added benefit of combining SN with gemcitabine in squamous pancreatic cancer cells. To date, there is no report of synergistic interaction between SN extracts when combined with gemcitabine in any cancer treatment study. A healthy individual will have no squamous cells in the pancreas. As compared to progenitor cells carcinoma, squamous cell carcinoma of the pancreas is associated with poor prognosis, more aggressive with higher metastasis rate and no targeted therapy [43, 44]. The major difference between squamous and progenitor carcinoma would be the transcription factors in endodermal development and differentiation. TGF-β and MYC were usually overexpressed among the squamous carcinoma, but not progenitor carcinoma [44]. In view of the upregulation of TGF-β among squamous carcinoma, gemcitabine treatment may down-regulate TGF-β in pancreatic tumor and resulting a better treatment outcomes achieved among the squamous carcinoma. The underlying mechanism behind gemcitabine inhibitory effect on TGF-β could be driven by its modulatory effect on STAT3 phosphorylation [45] Alternative explanation to the observed stronger synergism against squamous carcinoma may be attributed to MYC inhibitor in SN extracts (eg: fisetin [46]) may downregulate MYC to enhance the cytotoxicity effect of gemcitabine. Kim N et al. elucidated fisetin enhanced cytotoxicity effect of gemcitabine in squamous carcinoma via inhibition of MYC signalling [46]. Both possible explanations may require further phytochemistry studies and molecular mechanistic studies to identify the underlying mechanism. Nevertheless, the combination of SN extracts and gemcitabine may have more promising outcomes against pancreatic ductal carcinoma especially the squamous carcinoma. Current research has been revolving around discovering ways to target the molecular subtype as a roadmap for precision medicine in pancreatic cancer [47].

Our findings may encourage exploration of the role of nutraceuticals (SN extracts) in combining with pharmaceuticals (gemcitabine) for patients with squamous pancreatic adenocarcinoma. There have been several plant extracts found in the past which has synergistic interactions when used in combination with gemcitabine. Yu J and coworkers reported that the extract of Pao Pereira enhanced the anti-tumor effect of gemcitabine in mice bearing pancreatic tumors with reduced metastatic lesions in peritoneum [48]. The same research team also reported that *Rauwolfia vomitoria* extract can potentiate gemcitabine’s anti-tumor effects, via reduction in tumor burden and prevented metastatic potential in an orthotopic pancreatic cancer mouse model [49]. Alternative study by Archana Pandita and coworkers suggested that gemcitabine may synergise with betulinic acid and thymoquinone, both of which are common dietary compounds, by inhibiting the proliferation of pancreatic cancer cells, inducing apoptosis and down-regulating pyruvate kinase M2 isoforms [50]. Irofulven (a compound from sesquiterpene mushroom metabolites) [51], cucurbitaicin B (Cucurbitacae family) were some of the pure compounds isolated from plants that can enhance the anti-tumor effects of gemcitabine [52]. Not forgetting that, in the presence of SN extracts, we can reduce the dose of gemcitabine by 2.38–5.28 fold and maintain the effects of gemcitabine in pancreatic cancer cells. By reducing the dose of gemcitabine, we can potentially reduce the toxicities associated by gemcitabine [53–55]. Nanotechnology in formulation, such as niosomes or liposomes, may be able to co-deliver SN extract and gemcitabine together [56]. These studies supported that SN extracts could be used to potentiate a chemotherapy, and are worth further investigation.

It is known that bcl-2, cIAP-2, livin, survivin and XIAP are the anti-apoptotic markers that prevent cancer cells undergoing apoptosis [20, 22]. Thus, by suppressing these anti-apoptotic proteins, the combination of SN extracts and gemcitabine, may induce inhibition in cell proliferation and apoptosis. Particularly, high expression of these anti-apoptotic proteins is associated with higher risk of chemoresistance and poorer prognosis among cancer patients [57, 58]. Our data clearly shown that the downregulation of antiapoptotic proteins, particularly bcl-2, cIAP-2, and XIAP by the combination of SN extracts and gemcitabine could be probably enhancing the pro-apoptotic proteins, such as bax levels leading to apoptotic cell death. TLR-4, the innate immune receptor, is related to immune evasion and carcinogenesis [59]. A study shown TLR-4 were expressed in AsPC1, BxPC3, and SW1990 cells [60]. Surprisingly, even though the previous study [18] suggested that SN extracts can reduce TLR-4 activities, the level of TLR-4 in cancer cells was not affected by SN extracts or in combination with gemcitabine. These results are in favour of our hypothesis that SN extracts synergised the anticancer effects of gemcitabine via upregulation of pro-apoptotic proteins, and downregulating the antiapoptotic proteins, independent of TLR-4 expression. Since TLR-4 is an upstream innate immune receptor, extracts that do not inhibit TLR-4 expression may have less implication for the immune system of the host. It is important to maintain the immune system of the host, in view of the fact that most cancer patients may suffer immunosuppression due to the non-selective cytotoxicity of the chemotherapies.

The crux of the study is to provide evidence-based data to support or refute the traditional practices of *C. nutans* in cancer. Hence, we emphasised on phenotypic investigation, rather than detailed mechanistic investigation because the phenotypic observation or therapeutic response has yet to be established. To the best our knowledge, our results is the first to confidently suggest that *C. nutans* extracts should not be used alone or as a replacement to chemotherapy since the extracts were not potent in all the 23 human cancer cells. Since the study was only conducted only in vitro, future study should emphasis on its investigating the synergistic effects on pancreatic tumor animal model with detailed mechanistic study. The mechanistic study may further explore correlation of pro- and anti-apoptotic markers with hallmarks of cancer inflammatory markers such as TLR-4, p38, p65, pERK and pJNK. The animal study would also provide evidence whether the combination of SN extracts and gemcitabine may alter the tumor microenvironment and tumor-immune system interaction. Also, it would be useful to determine the active metabolites from SN extracts. Our findings suggested the commonly isolated compounds (such as vitexin and isovitexin) had no anti-cancer effects. It is strongly believe that other active metabolites in SN extracts have yet to be uncovered. Detailed chromatography studies would be of useful to isolate and to characterise the active metabolites. With the known active metabolites isolated and characterised, it would be clearer to determine the pharmacodynamic and pharmacokinetics interaction between the active metabolites and gemcitabine. It may provide new evidence for future study to investigate the potential of these metabolites in enhance chemosensitivity and to reverse chemoresistance in human pancreatic cancers.

## Conclusion

Our results provide confidence that *C. nutans* extracts should not be administered to patients as a replacement to chemotherapy. However, the findings support the development of SN extracts to synergise the antitumor effects of gemcitabine in squamous pancreatic ductal adenocarcinoma. The concentration of gemcitabine required to achieve its median effective dose in pancreatic cancer treatment, could be reduced by 2.38- to 5.28-fold. Further studies may concentrate on the active metabolites of SN extracts as well as determining the synergised mechanism of actions in squamous pancreatic cancer animal studies.

## Additional file

Additional file 1: **Figure S1.** The matrix plots from Combenefit reported the HSA and Bliss synergy/antagonism score ± standard deviation for each combination treatment of SN and gemcitabine at different concentrations. Each combination was colored according to the scale where red and blue represent antagonism and synergy, respectively and the asterisk(s) indicate the level of significance (* *p* < 0.05, ** *p* < 0.001, *** *p* < 0.0001). **Table S1.** Description of Combination Index in Chou-Talalay method **Table S2.** Description of Bliss interaction volume in MacSynergy™ II. **Table S3.** Dose reduction index of gemcitabine and SN combinatorial treatment at different ratios on PDAC cells. **Table S4.** IC50 of Pure Compounds in *C. nutans* Extracts. (DOCX 1089 kb)

## Data Availability

All data generated or analysed during this study are included in the supporting information and in the published article.

## Abbreviations

ANOVA: One-way analysis of variance
Bax: Bcl-2-associated X protein
Bcl-2: B-cell lymphoma 2
BID: Bcl-2 homology 3 interacting domain death agonist
CI: Combination index
cIAP-2: Cellular inhibitor of apoptosis 2
DMSO: Dimethylsulfoxide
DRI: Drug reduction index
ELISA: Enzyme-linked immunosorbent assay
EOHSA: Excess Over Highest Single Agent
Gem: Gemcitabine
HSA: Highest Single Agent
IC_50_: 50% inhibitory concentration
LN: Non-polar leaf extracts of *Clinacanthus nutans*
LP: Polar leaf extracts of *Clinacanthus nutans*
MTT: 3-(4,5-dimethylthiazol-2-yl)-2,5-diphenyltetrazolium bromide
PDAC: Pancreatic ductal adenocarcinoma
SN: Non-polar stem extracts of *Clinacanthus nutans*
SP: Polar stem extracts of *Clinacanthus nutans*
TFC: Total flavonoid content
TLR-4: Toll-like receptor 4
TNF: Tumor necrosis factor
TPC: Total phenolic content
TRAILR: Tumor necrosis factor related apoptosis inducing ligand
XIAP: X-linked inhibitor of apoptosis protein

## References

[1] Brantley SJ, Argikar AA, Lin YS, Nagar S, Paine MF (2014). Herb-drug interactions: challenges and opportunities for improved predictions. Drug Metab Dispos.

[2] Fasinu PS, Bouic PJ, Rosenkranz B (2012). An overview of the evidence and mechanisms of herb-drug interactions. Front Pharmacol.

[3] Wu YS, Lee ZY, Chuah LH, Mai CW, Ngai SC (2019). Epigenetics in metastatic breast cancer: its regulation and implications in diagnosis, prognosis and therapeutics. Curr Cancer Drug Targets.

[4] Mai CW, Chung FF, Leong CO (2017). Targeting Legumain as a novel therapeutic strategy in cancers. Curr Drug Targets.

[5] Chung FF, Mai CW, Ng PY, Leong CO (2016). Cytochrome P450 2W1 (CYP2W1) in colorectal cancers. Curr Cancer Drug Targets.

[6] Tham SY, Loh HS, Mai CW, Fu JY (2019). Tocotrienols modulate a life or death decision in cancers. Int J Mol Sci.

[7] Krishnan Premanand, Lee Fong-Kai, Chong Kam-Weng, Mai Chun-Wai, Muhamad Azira, Lim Siew-Huah, Low Yun-Yee, Ting Kang-Nee, Lim Kuan-Hon (2018). Alstoscholactine and Alstolaxepine, Monoterpenoid Indole Alkaloids with γ-Lactone-Bridged Cycloheptane and Oxepane Moieties from Alstonia scholaris. Organic Letters.

[8] Krishnan P, Mai CW, Yong KT, Low YY, Lim KH (2019). Alstobrogaline, an unusual pentacyclic monoterpenoid indole alkaloid with aldimine and aldimine-N-oxide moieties from Alstonia scholaris. Tetrad Lett.

[9] Al-Khdhairawi AAQ, Krishnan P, Mai CW, Chung FF, Leong CO, Yong KT (2017). A Bis-benzopyrroloisoquinoline alkaloid incorporating a Cyclobutane Core and a Chlorophenanthroindolizidine alkaloid with cytotoxic activity from Ficus fistulosa var. tengerensis. J Nat Prod.

[10] Chung FF, Tan PF, Raja VJ, Tan BS, Lim KH, Kam TS, et al. Jerantinine a induces tumor-specific cell death through modulation of splicing factor 3b subunit 1 (SF3B1). Sci Rep. 2017;7:42504. 10.1038/srep42504. 10.1038/srep42504 PMC5309811 28198434

[11] Soo HC, Chung FF, Lim KH, Yap VA, Bradshaw TD, Hii LW (2017). Cudraflavone C induces tumor-specific apoptosis in colorectal cancer cells through inhibition of the phosphoinositide 3-kinase (PI3K)-AKT pathway. PLoS One.

[12] Krishnan P, Mai C-W, Yong K-T, Low Y-Y, Lim K-H (2019). Alstobrogaline, an unusual pentacyclic monoterpenoid indole alkaloid with aldimine and aldimine-N-oxide moieties from *Alstonia scholaris*. Tetrahedron Lett.

[13] Sakdarat S, Shuyprom A, Pientong C, Ekalaksananan T, Thongchai S (2009). Bioactive constituents from the leaves of Clinacanthus nutans Lindau. Bioorg Med Chem.

[14] Charuwichitratana S, Wongrattanapasson N, Timpatanapong P, Bunjob M (1996). Herpes zoster: treatment with Clinacanthus nutans cream. Int J Dermatol.

[15] Yong YK, Tan JJ, Teh SS, Mah SH, Ee GC, Chiong HS (2013). Clinacanthus nutans extracts are antioxidant with Antiproliferative effect on cultured human cancer cell lines. Evid Based Complement Alternat Med.

[16] Liew SY, Stanbridge EJ, Yusoff K, Shafee N (2012). Hypoxia affects cellular responses to plant extracts. J Ethnopharmacol.

[17] Houghton P, Fang R, Techatanawat I, Steventon G, Hylands PJ, Lee CC (2007). The sulphorhodamine (SRB) assay and other approaches to testing plant extracts and derived compounds for activities related to reputed anticancer activity. Methods..

[18] Mai CW, Yap KS, Kho MT, Ismail NH, Yusoff K, Shaari K (2016). Mechanisms underlying the anti-inflammatory effects of Clinacanthus nutans Lindau extracts: inhibition of cytokine production and toll-like Receptor-4 activation. Front Pharmacol.

[19] Tan BS, Kang O, Mai CW, Tiong KH, Khoo AS, Pichika MR (2013). 6-Shogaol inhibits breast and colon cancer cell proliferation through activation of peroxisomal proliferator activated receptor gamma (PPARgamma). Cancer Lett.

[20] Mai CW, Kang YB, Nadarajah VD, Hamzah AS, Pichika MR (2018). Drug-like dietary vanilloids induce anticancer activity through proliferation inhibition and regulation of bcl-related apoptotic proteins. Phytother Res.

[21] Yeong KY, Tan SC, Mai CW, Leong CO, Chung FF, Lee YK (2018). Contrasting sirtuin and poly (ADP-ribose) polymerase activities of selected 2,4,6-trisubstituted benzimidazoles. Chem Biol Drug Des.

[22] Mai CW, Yaeghoobi M, Abd-Rahman N, Kang YB, Pichika MR (2014). Chalcones with electron-withdrawing and electron-donating substituents: anticancer activity against TRAIL resistant cancer cells, structure-activity relationship analysis and regulation of apoptotic proteins. Eur J Med Chem.

[23] Chung FF, Tan PF, Raja VJ, Tan BS, Lim KH, Kam TS (2017). Jerantinine a induces tumor-specific cell death through modulation of splicing factor 3b subunit 1 (SF3B1). Sci Rep.

[24] Chou TC, Talalay P (1984). Quantitative analysis of dose-effect relationships: the combined effects of multiple drugs or enzyme inhibitors. Adv Enzym Regul.

[25] Er JL, Goh PN, Lee CY, Tan YJ, Hii LW, Mai CW (2018). Identification of inhibitors synergizing gemcitabine sensitivity in the squamous subtype of pancreatic ductal adenocarcinoma (PDAC). Apoptosis..

[26] Low SY, Tan BS, Choo HL, Tiong KH, Khoo AS, Leong CO (2012). Suppression of BCL-2 synergizes cisplatin sensitivity in nasopharyngeal carcinoma cells. Cancer Lett.

[27] Wong SW, Tiong KH, Kong WY, Yue YC, Chua CH, Lim JY (2011). Rapamycin synergizes cisplatin sensitivity in basal-like breast cancer cells through up-regulation of p73. Breast Cancer Res Treat.

[28] Chou TC (2006). Theoretical basis, experimental design, and computerized simulation of synergism and antagonism in drug combination studies. Pharmacol Rev.

[29] Di Veroli GY, Fornari C, Wang D, Mollard S, Bramhall JL, Richards FM (2016). Combenefit: an interactive platform for the analysis and visualization of drug combinations. Bioinformatics..

[30] Greco WR, Bravo G, Parsons JC (1995). The search for synergy: a critical review from a response surface perspective. Pharmacol Rev.

[31] Keith CT, Borisy AA, Stockwell BR (2005). Multicomponent therapeutics for networked systems. Nat Rev Drug Discov.

[32] Prichard MN, Shipman C (1990). A three-dimensional model to analyze drug-drug interactions. Antivir Res.

[33] Eisenberg DM, Davis RB, Ettner SL, Appel S, Wilkey S, Van Rompay M (1998). Trends in alternative medicine use in the United States, 1990-1997: results of a follow-up national survey. JAMA..

[34] Engel LW, Straus SE (2002). Development of therapeutics: opportunities within complementary and alternative medicine. Nat Rev Drug Discov.

[35] Astin JA (1998). Why patients use alternative medicine: results of a national study. JAMA..

[36] Johnson SB, Park HS, Gross CP, Yu JB (2018). Complementary medicine, refusal of conventional cancer therapy, and survival among patients with curable cancers. JAMA Oncol.

[37] Richardson MA, Sanders T, Palmer JL, Greisinger A, Singletary SE (2000). Complementary/alternative medicine use in a comprehensive cancer center and the implications for oncology. J Clin Oncol.

[38] Dy GK, Bekele L, Hanson LJ, Furth A, Mandrekar S, Sloan JA (2004). Complementary and alternative medicine use by patients enrolled onto phase I clinical trials. J Clin Oncol.

[39] Vapiwala N, Mick R, Hampshire MK, Metz JM, DeNittis AS (2006). Patient initiation of complementary and alternative medical therapies (CAM) following cancer diagnosis. Cancer J.

[40] Looi CK, Chung FFL, Leong CO, Wong SF, Rosli R, Mai CW (2019). Therapeutic challenges and current immunomodulatory strategies in targeting the immunosuppressive pancreatic tumor microenvironment. J Exp Clin Cancer Res.

[41] Zulkipli IN, Rajabalaya R, Idris A, Sulaiman NA, David SR (2017). Clinacanthus nutans: a review on ethnomedicinal uses, chemical constituents and pharmacological properties. Pharm Biol.

[42] Khoo LW, Audrey Kow S, Lee MT, Tan CP, Shaari K, Tham CL (2018). A comprehensive review on Phytochemistry and pharmacological activities of Clinacanthus nutans (Burm.F.) Lindau. Evid Based Complement Alternat Med.

[43] Bailey P, Chang DK, Nones K, Johns AL, Patch AM, Gingras MC (2016). Genomic analyses identify molecular subtypes of pancreatic cancer. Nature..

[44] Camolotto SA, Belova VK, Snyder EL (2018). The role of lineage specifiers in pancreatic ductal adenocarcinoma. J Gastrointest Oncol.

[45] Eriksson E, Wenthe J, Irenaeus S, Loskog S, Ullenhag G (2016). Gemcitabine reduces MDSCs, tregs and TGFβ-1 while restoring the teff/treg ratio in patients with pancreatic cancer. J Transl Med.

[46] Kim N, Kang MJ, Lee SH, Son JH, Lee JE, Paik WH (2018). Fisetin enhances the cytotoxicity of gemcitabine by down-regulating ERK-MYC in MiaPaca-2 human pancreatic cancer cells. Anticancer Res.

[47] Grippo PJ (2018). Pancreatic cancer subtypes: a roadmap for precision medicine AU - Torres, Carolina. Ann Med.

[48] Yu J, Drisko J, Chen Q (2013). Inhibition of pancreatic cancer and potentiation of gemcitabine effects by the extract of Pao Pereira. Oncol Rep.

[49] Yu J, Chen Q (2014). Antitumor activities of Rauwolfia vomitoria extract and potentiation of gemcitabine effects against pancreatic cancer. Integr Cancer Ther.

[50] Pandita A, Kumar B, Manvati S, Vaishnavi S, Singh SK, Bamezai RNK (2014). Synergistic combination of gemcitabine and dietary molecule induces apoptosis in pancreatic cancer cells and down regulates PKM2 expression. PLoS One.

[51] Van Laar ES, Roth S, Weitman S, Macdonald JR, Waters SJ (2004). Activity of Irofulven against human pancreatic carcinoma cell lines in vitro and in vivo. Anticancer Res.

[52] Yue Q, Gao G, Zou G, Yu H, Zheng X (2017). Natural products as adjunctive treatment for pancreatic cancer: recent trends and advancements. Biomed Res Int.

[53] Venat-Bouvet L, Ly K, Szelag JC, Martin J, Labourey JL, Genet D (2003). Thrombotic microangiopathy and digital necrosis: two unrecognized toxicities of gemcitabine. Anti-Cancer Drugs.

[54] Teusink AC, Hall PD (2010). Toxicities of gemcitabine in patients with severe hepatic dysfunction. Ann Pharmacother.

[55] Matsubara J, Ono M, Negishi A, Ueno H, Okusaka T, Furuse J (2009). Identification of a predictive biomarker for hematologic toxicities of gemcitabine. J Clin Oncol.

[56] Maniam G, Mai CW, Zulkefeli M, Dufes C, Tan DM, Fu JY (2018). Challenges and opportunities of nanotechnology as delivery platform for Tocotrienols in cancer therapy. Front Pharmacol.

[57] Sharma J, Srinivasan R, Majumdar S, Mir S, Radotra BD, Wig JD (2005). Bcl-XL protein levels determine apoptotic index in pancreatic carcinoma. Pancreas..

[58] Ferrandina G, Legge F, Martinelli E, Ranelletti FO, Zannoni GF, Lauriola L (2005). Survivin expression in ovarian cancer and its correlation with clinico-pathological, surgical and apoptosis-related parameters. Br J Cancer.

[59] Mai CW, Kang YB, Pichika MR (2013). Should a toll-like receptor 4 (TLR-4) agonist or antagonist be designed to treat cancer? TLR-4: its expression and effects in the ten most common cancers. Onco Targets Ther.

[60] Vaz J, Andersson R (2014). Intervention on toll-like receptors in pancreatic cancer. World J Gastroenterol.

