# Supplementary material for: The synergism of Clinacanthus nutans Lindau extracts with gemcitabine: downregulation of anti-apoptotic markers in squamous pancreatic ductal adenocarcinoma
Source: BMC Complement Altern Med. 2019 Sep 14;19:257. doi: 10.1186/s12906-019-2663-9 (PMC6744713; doi:10.1186/s12906-019-2663-9)
Supplement: Supplementary file 1 — Figure S1. The matrix plots from Combenefit reported the HSA and Bliss synergy/antagonism score ± standard deviation for each combination treatment of SN and gemcitabine at different concentrations. Each combination was colored according to the scale where red and blue represent antagonism and synergy, respectively and the asterisk(s) indicate the level of significance (* p < 0.05, ** p < 0.001, *** p < 0.0001). Table S1. Description of Combination Index in Chou-Talalay method Table S2. Description of Bliss interaction volume in MacSynergy™ II. Table S3. Dose reduction index of gemcitabine and SN combinatorial treatment at different ratios on PDAC cells. Table S4. IC50 of Pure Compounds in C. nutans Extracts. (DOCX 1089 kb) [file 12906_2019_2663_MOESM1_ESM.docx]

**Additional file 1**


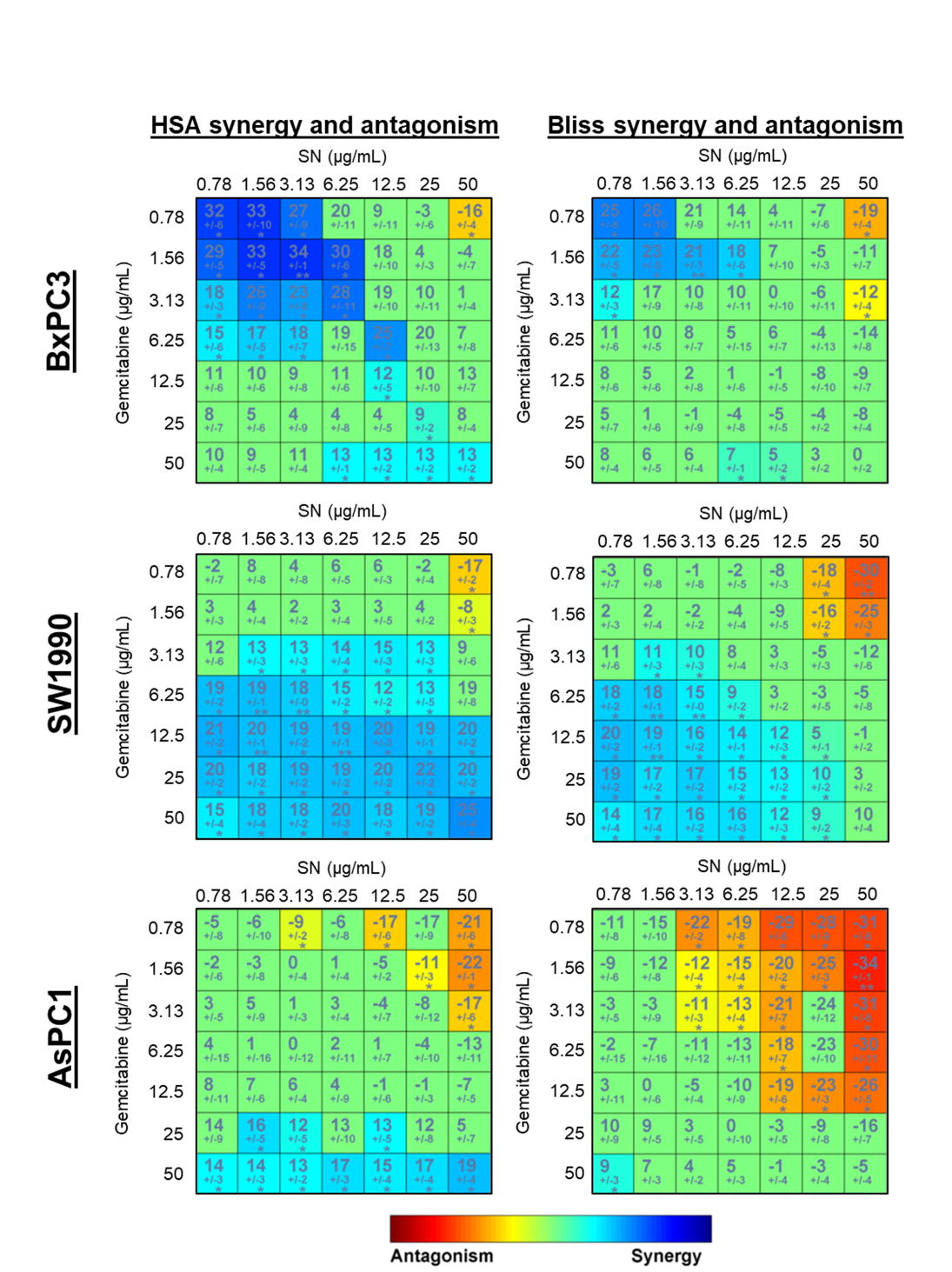


**Figure S1. The Matrix Plots from Combenefit Reported the HSA and Bliss Synergy/Antagonism Score ± Standard Deviation for Each Combination Treatment of SN and Gemcitabine at Different Concentrations**. Each combination was colored according to the scale where red and blue represent antagonism and synergy, respectively and the asterisk(s) indicate the level of significance (* p< 0.05, ** p<0.001, *** p<0.0001).

**Table S1. Description of Combination Index in Chou-Talalay Method.**

| **Range of Combination Index** | **Description** |
| --- | --- |
| < 0.1 | Very strong synergism |
| 0.10 – 0.30 | Strong synergism |
| 0.30 – 0.70 | Synergism |
| 0.70 – 0.85 | Moderate synergism |
| 0.85 – 0.90 | Slight synergism |
| 0.90 – 1.10 | Nearly additive |
| 1.10 – 1.20 | Slight antagonism |
| 1.20 – 1.45 | Moderate antagonism |
| 1.45 – 3.30 | Antagonism |
| 3.30 – 10 | Strong antagonism |
| > 10 | Very strong antagonism |

**Table S2. Description of Bliss Interaction Volume in MacSynergy^TM^ II.**

| **Volume (μM^2^%)** | **Description** |
| --- | --- |
| < – 100 | Strong Bliss antagonism and probably important *in vivo*. |
| ≤ – 50 | Moderate Bliss antagonism. This interaction may be important *in vivo*. |
| < – 25 | Minor but significant amount Bliss antagonism. |
| – 25 to 25 | Bliss additivity and probably insignificant interaction. |
| > 25 | Minor but significant amount Bliss synergy. |
| ≥ 50 | Moderate Bliss synergy. This interaction may be important *in vivo*. |
| > 100 | Strong Bliss synergy and probably important *in vivo*. |

**Table S3. Dose Reduction Index of Gemcitabine and SN Combinatorial Treatment at Different Ratios on PDAC**

|  | | | | | | | | | | | | |
| --- | --- | --- | --- | --- | --- | --- | --- | --- | --- | --- | --- | --- |
| **Cell Lines** | **Gem*:SN ratio** | **Dose Reduction Index of Gemcitabine** | | | | |  | **Dose Reduction Index of SN Extracts** | | | | |
|  |  | **Fa=0.50** | **Fa=0.75** | **Fa=0.90** | **Mean** | **±SD** |  | **Fa=0.50** | **Fa=0.75** | **Fa=0.90** | **Mean** | **±SD** |
| **BxPC3** | **1:4** | 4.01 | 2.46 | 1.51 | 2.66 | ± 1.26 |  | 4.40 | 5.16 | 6.04 | 5.20 | ± 0.82 |
|  | **1:2** | 4.94 | 3.06 | 1.89 | 3.29 | ± 1.54 |  | 10.84 | 12.82 | 15.16 | 12.94 | ± 2.16 |
|  | **1:1** | 4.67 | 2.65 | 1.51 | 2.95 | ± 1.60 |  | 20.53 | 22.27 | 24.16 | 22.32 | ± 1.81 |
|  | **2:1** | 3.65 | 2.34 | 1.51 | 2.50 | ± 1.08 |  | 32.02 | 39.32 | 48.28 | 39.88 | ± 8.14 |
|  | **4:1** | 2.38 | 2.11 | 1.86 | 2.12 | ± 0.26 |  | 41.86 | 70.70 | 119.41 | 77.32 | ± 39.20 |
| **SW1990** | **1:4** | 3.71 | 4.98 | 6.69 | 5.13 | ± 1.50 |  | 4.40 | 5.40 | 6.62 | 5.47 | ± 1.11 |
|  | **1:2** | 4.31 | 5.55 | 7.16 | 5.67 | ± 1.43 |  | 10.23 | 12.03 | 14.16 | 12.14 | ± 1.97 |
|  | **1:1** | 3.92 | 5.14 | 6.74 | 5.27 | ± 1.42 |  | 18.61 | 22.28 | 26.67 | 22.52 | ± 4.04 |
|  | **2:1** | 4.16 | 4.14 | 4.12 | 4.14 | ± 0.02 |  | 39.53 | 35.90 | 32.60 | 36.01 | ± 3.47 |
|  | **4:1** | 5.28 | 3.84 | 2.79 | 3.97 | ± 1.25 |  | 100.31 | 66.53 | 44.12 | 70.32 | ± 28.28 |
| **AsPC1** | **1:4** | 4.27 | 12.71 | 37.87 | 18.28 | ± 17.48 |  | 1.13 | 1.26 | 1.41 | 1.27 | ± 0.14 |
|  | **1:2** | 4.92 | 14.47 | 42.58 | 20.66 | ± 19.58 |  | 2.60 | 2.87 | 3.17 | 2.88 | ± 0.28 |
|  | **1:1** | 4.28 | 12.53 | 36.70 | 17.84 | ± 16.85 |  | 4.53 | 4.97 | 5.46 | 4.99 | ± 0.47 |
|  | **2:1** | 4.36 | 11.16 | 28.57 | 14.69 | ± 12.48 |  | 9.22 | 8.85 | 8.50 | 8.85 | ± 0.36 |
|  | **4:1** | 4.14 | 9.48 | 21.71 | 11.78 | ± 9.01 |  | 17.50 | 15.04 | 12.92 | 15.15 | ± 2.29 |

*Gem = Gemcitabine; SN = SN Extracts

**Table S4. IC_50_ of Pure Compounds in *C. nutans* Extracts**

| **Cell Line** | **AsPC1** | **BxPC3** | **SW1990** | **MRC5** | **ARPE19** |
| --- | --- | --- | --- | --- | --- |
| Betulin | >100 | >100 | >100 | >100 | >100 |
| Lupeol | >100 | >100 | >100 | >100 | >100 |
| Beta-sitosterol | >100 | >100 | >100 | >100 | >100 |
| Isovitexin | >100 | >100 | >100 | >100 | >100 |
| Vitexin | >100 | >100 | >100 | >100 | >100 |
| Rutin | >100 | >100 | >100 | >100 | >100 |
| Chlorogenic acid | >100 | >100 | >100 | >100 | >100 |
| Gallic acid | >100 | >100 | >100 | >100 | >100 |
